# Fuzzy inference game approach to uncertainty in business decisions and market competitions

**DOI:** 10.1186/2193-1801-2-484

**Published:** 2013-09-25

**Authors:** Festus Oluseyi Oderanti

**Affiliations:** Plymouth Business School, University of Plymouth, Plymouth, PL4 8AA UK

**Keywords:** Fuzzy logic, Membership functions, Zero sum, Decision, Business games, Game theory

## Abstract

The increasing challenges and complexity of business environments are making business decisions and operations more difficult for entrepreneurs to predict the outcomes of these processes. Therefore, we developed a decision support scheme that could be used and adapted to various business decision processes. These involve decisions that are made under uncertain situations such as business competition in the market or wage negotiation within a firm. The scheme uses game strategies and fuzzy inference concepts to effectively grasp the variables in these uncertain situations. The games are played between human and fuzzy players. The accuracy of the fuzzy rule base and the game strategies help to mitigate the adverse effects that a business may suffer from these uncertain factors. We also introduced learning which enables the fuzzy player to adapt over time. We tested this scheme in different scenarios and discover that it could be an invaluable tool in the hand of entrepreneurs that are operating under uncertain and competitive business environments.

## Introduction

A decision is a (conscious) choice of a move (or action) from among a well-defined set of alternatives and an individual decision maker is motivated to act in such a manner that the expected value to him of the outcome is as high as possible (Shubik 1958). The individual decision maker can attach a value to the outcomes arising from any set of moves.

The decision maker is an individual at the simplest level and a decision process must have a purpose in so far as it only exists to further a particular objective or goal of the decision maker (Oderanti 2010). When faced with certain problems, an individual rational decision maker will make attempts to order or rank his goals or objectives in some certain relative order. The decision maker will then be in a position to examine various alternative means in order to achieve the desired goals. He will then choose the best strategy which either minimizes the costs of any possible failure or maximizes the set objectives to achieve the desired goals (McGrew and Wilson 1982).

Moreover, a decision maker is frequently confronted with fuzzy constraints, fuzzy utility maximization, and fuzziness about the state of competitors (De Wilde 2004). There are many decision situations when we cannot process the information contained precisely in a quantitative form but which may need to be rather accessed or processed in qualitative form and therefore, the need for us to adopt a *linguistic approach* (Herrera and Viedma 2000). Decision-makers in a conflict often make their decisions under risk and under unclear or fuzzy information (Li et al. 2001).

This research aims at developing an efficient decision support scheme simulated in the form of a non-cooperative zero-sum game with imperfect information, using fuzzy logic concepts that can assist a business organization in making an effective decision in a competitive market environment. We used a general illustration to describe the model and we have verified the validity of our results with more case studies and real data in our other papers in (Oderanti and De Wilde 2010; Oderanti and Wilde 2011; Oderanti et al. 2012).

The contribution of this paper is that it illustrates how an entrepreneur could make effective and efficient business decisions by using fuzzy inference systems (FIS) in capturing uncertainties that may surround his business environments. This will help the entrepreneur to have competitive advantages over his competitors. The models also include learning procedures that enable the agents to optimize the fuzzy rules and their decision processes. This is another contribution of the paper: a set of fuzzy models that include learning, and can be used to improve decision making in business.

## Fuzzy logic and fuzzy decision making system

As the complexity of a system increases, the utility of fuzzy logic as a modeling tool increases (Oderanti 2010). For very complex systems, few numerical data may exist and only ambiguous and imprecise information and knowledge is available. Fuzzy logic allows approximate interpolation between input and output situations (Keshwani et al. 2008).

Fuzzy logic provides a framework that attempts to define a natural way of dealing with problems in which the source of imprecision is the absence of sharply defined criteria of class membership rather than the presence of random variables (Zadeh 1965). It is a problem solving technique that was introduced by Lotfi Zadeh in (Zadeh 1965) to deal with vague or imprecise problems (De Wilde 2004; DeWilde 2002; Dweiri and Kablan 2006; Kandel and Zhang 1998). It is used to model human reasoning and knowledge that do not have well defined boundary. Although fuzzy logic covers a wide range of theories and techniques, it is mainly based on four concepts: fuzzy sets, linguistic variables (Hajjaji and Rachid 1995), possibility distributions (membership functions), and fuzzy if-then-rules (Dweiri and Kablan 2006). The values of a linguistic variable are both quantitatively described by a fuzzy set. Possibility distributions or membership functions are constraints on the value of a linguistic variable imposed by assigning it a fuzzy set. Fuzzy if then rules are knowledge representation schemes for describing a functional mapping between antecedents and consequents (Oderanti 2010). A fuzzy inference system employs fuzzy if-then rules and can model the qualitative aspects of human knowledge and reasoning processes without employing precise quantitative analysis (Oderanti 2010). Fuzzy inference systems are generally understandable because the knowledge in these systems is contained in the form of fuzzy if-then rules containing membership functions (Anderson and Hall 1999).

**Figure 1:**
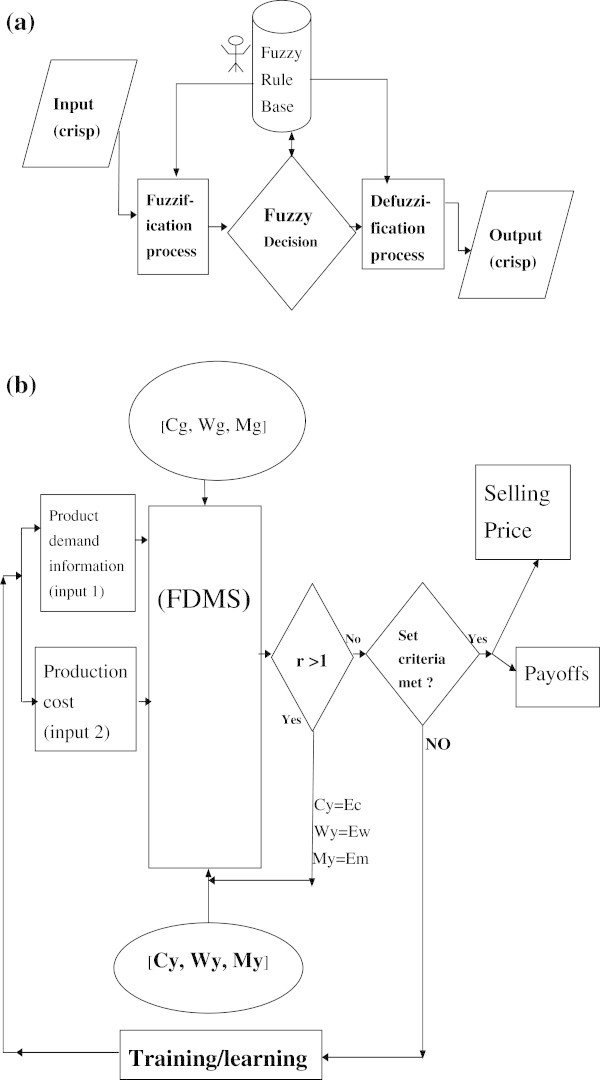
**The decision model for the fuzzy inference system.** **(a)** Fuzzy decision making system (FDMS), **(b)** fuzzy inference system for business decisions (FISBD) model.

In general, a fuzzy decision making system (FDMS) uses a collection of fuzzy membership functions and decision rules that are solicited from experts in the field to reason about data (Dweiri and Kablan 2006). The components of an FDMS contain a fuzzification section, a fuzzy rule base, fuzzy decision logic and a defuzzification section as shown in Figure 1a.

### Sources of fuzzy rules

As in many applications of fuzzy rule-based systems, the fuzzy if-then rules used in our models have been solicited from human experts (Nozaki et al. 1997; Chen et al. 2008). We sought knowledge from human experts in the fields that are related to each scenario. In all the simulations, the accuracy of these solicited rules are judged and amended by searching related data from published economic and fuzzy inference literatures such as (Griffiths and Wall 2000; Himmelweit et al. 2001; Oderanti and De Wilde 2010; Ross 2005; Negnevitsky 2005).

## Game theory

### Game theory framework

Game theory has had a deep impact on the theory of industrial organization (Fisher 1989). The reason it has been embraced by a majority of researchers in the field is that it imposes some discipline on theoretical thinking (Oderanti 2010). It forces economists to clearly specify the strategic variables, their timing, and the information structure faced by firms (Oderanti 2010).

Game theory is a method for the study of decision-making in situations of conflict and it deals with problems in which the individual decision-maker is not in complete control of the factors influencing the outcome (Shubik 1955). It was developed to quantify, model and explain human behavior under conflicts between individuals and public interests (Lee et al. 2008). A decision-maker in a game faces a cross-purposes maximization problem. He must plan for an optimal return, taking into account the possible actions of his opponents. A game is a model of a situation where two or more groups are in dispute over some issues or resources (Li et al. 2001). A player in a game is an autonomous decision-making unit. Tapan Biswas in (Tapan 1997) also stated that a large part of the decision making processes under uncertainties can be covered by game theory. It deals with decision-making processes involving two or more parties, also known as players with partly or completely conflicting interest (Li et al. 2001; Shubik 1955) and it is one of the methodologies designed for application to the social sciences. All situations in which at least one agent can only act to maximize his utility through anticipating (either consciously, or just implicitly in his behaviour) the responses to his actions by one or more other agents are called games and agents involved in games are referred to as players (Ross 2010; De Wilde 2004) and could represent people, military, firms, countries or other organisations (Braathen and Sendstad 2004; Li et al. 2001).

### Business games

A business simulation or game may be defined as a sequential decision-making exercise structured around a model of a business operation in which participants assume the role of managing the simulated operation (Shubik 1972). A business game is a contrived situation which imbeds players in a simulated business environment, where they must make management-type decisions from time to time, and their choices at one time generally affect the environmental conditions under which the subsequent decisions must be made. Further, the interactions between decision and environment are determined by a refereeing process which is not open to argument from the players (Oderanti 2010).

In business games, the firm identifies the moves that the rival could make in response to each of its strategies. The firm can then plan counter-strategies (Griffiths and Wall 2000). As Doug Ivester, Coca-Cola’s president put it (Himmelweit et al. 2001) "I look at the business like a chessboard. You always need to be seeing three, four, five moves ahead; otherwise, your first move can prove fatal". Game theory (De Wilde 2004; Li et al. 2001; Ross 2010; Braathen and Sendstad 2004; Kandel and Zhang 1998) helps explore the impact of calculations about future market advantages on a firm’s current market strategies.

In business games, the conflicting interest of a firm may be to minimize the cost function, maximize the market share, or maximize the profit (Li et al. 2001). In this game, profit maximization of the fuzzy player is to be achieved through learning by the fuzzy player, and minimization of the payoffs of the opponents.

## Players’ strategies

A strategy is a decision rule that specifies how the player will act in every possible circumstance (Oderanti and De Wilde 2010). It is a specific course of action taken by the firm. This will involve the firm allocating values to its policy variables. These policy variables are generally those aspects of its activities that the firm can directly affect and may include price, spending on promotion, marketing, research and development and so on. For each strategy of this firm, its rival (or rivals) may adopt counter-strategies (Griffiths and Wall 2000).

The outcome of a game will depend upon the strategies employed by every player. In games, any pure strategy, which can be rejected by comparing it with the other pure strategies and finding that there are others which are always better under every circumstance, is a dominated strategy and will not enter into a solution (Shubik 1955).

In our experiment, each player is given five units of initial resources which may represent capital, time, personnel or other business resources. In this case study, we assume capital (say £5M). In each round, the players may choose to allocate their resources to one of three roles: *consolidation efforts* (*C*), *reserved or generated wealth* (*W*) and *aggressive marketing efforts* (*M*). These resource allocations will be done simultaneously by both players. Only the opponent’s move history will be known, but without knowledge of the opponent’s current choice of strategy. The allocations are denoted as a vector [ *C*,*W*,*M*] for each player and constitute the *strategy* of that player.

Consolidation efforts *C* refer to the proportion of resources that are spent to retain existing customers (if any) such as various customer service improvements, customer care, satisfaction, delight and customer retention initiatives. Marketing aggressiveness *M* denotes the part of these resources that are allocated to various advertising, marketing and promotional campaigns. These are principally targeted towards getting new customers. Reserved wealth *W* refers to part of the resources that are kept unused in the firm’s coffer.

As examples of players’ strategies, consider a firm **Y** that is a new entrant into a market. **Y** does not have existing customers to consolidate at the start of the game and therefore has *C* = 0. It may then decide to allocate all or most of its resources on advertising (marketing) campaigns *M*. If it chooses to allocate all to marketing *M* = 5, then its strategy [ *C*_*y*_,*W*_*y*_,*M*_*y*_] becomes [ 0,0,5]. This is considered to be the strongest strategy. We refer to it in Section "Fuzzy inference system for business decisions (FISBD)" Step 11, as globally optimizing player (*Geq*).

Assume **Y** enters the market with a much reduced price *E*_*sp*_ (probably as a result of new technology which leads to reduced production cost *C*_*p*_). If it is economically impossible for the incumbent (existing) firm **G** to cut its price to the same level due to its high production cost *C*_*p*_, **G** may decide to devote most of its resources (say £4M) to consolidate its existing customers in order to retain its market share. It may then decide to allocate the remaining resources *M*_*g*_ to market new customers. Therefore, **G**’s strategy [ *C*_*g*_,*W*_*g*_,*M*_*g*_] becomes [ 4,0,1].

The difference between strategies of different players is the proportion, number or amount that each player decides to allocate to each component of his own strategy [ *C*,*W*,*M*] out of his available total resources (say £5M). This is how firms allocate resources to their core strategies for competition (such as advertisement) and how these allocations could affect their payoffs in an uncertain or fuzzy market environment.

The variables in our models can be tailored to the business situations in the real world and therefore are not limited to those variables that we have used in designing the system. Therefore, this model can be applied to any real business situation and the variables can be adapted to suit the situation in question.

The model can also work for systems that have more strategic variables than those that we have used in this model.

## Fuzzy inference system for business decisions (FISBD)

**Figure 2:**
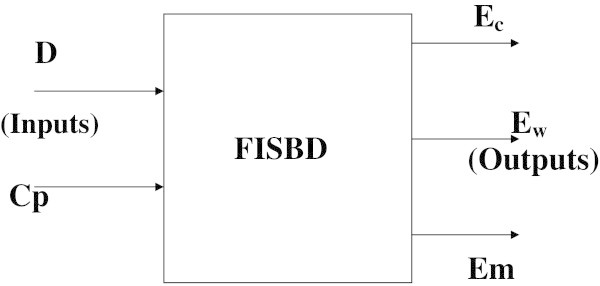
**A basic model of the FISBD engine showing the two inputs** ***D*** **,** ***C***_***p***_ **and the three outputs** ***E***_***c***_ **,** ***E***_***w***_ **and** ***E***_***m***_ **.**

The model for our proposed fuzzy inference system for business decisions (FISBD) is as shown in Figure 1(b) while a simplified version of the figure is as shown in Figure 2.

Our FISBD involves two players (firms) in a typical duopoly market which we shall represent as green (*g*) and yellow (*y*) which represents the *fuzzy agent*. Each player is given five units of initial resources which may represent capital, time, personnel or other business resources. In our case we assume capital (say £5M). The number of rounds the game must be played is five which denotes a sequence of five possible moves for each player. In each round, the players may choose to allocate their units between three roles (strategies): *consolidation efforts* (*C*), *reserved or generated wealth* (*W*) and *aggressive marketing efforts* (*M*). These resources allocation will be done simultaneously with only the opponent move history that will be known but without knowledge of the opponent’s current choice of strategy and are denoted as vector [ *C*,*W*,*M*] for each player.

The general procedures necessary for designing the proposed decision support system (FISBD) are as listed in the steps below:

1. List all uncertain (fuzzy) factors that will be considered in taking the business decision: the uncertain or fuzzy information (factors) we are taking into consideration in this illustration are anticipated market demand information (*D*) and the production costs(*C*_*P*_).
2. Determine the strategies of the players: Here, we are adopting three strategies for each player and these strategies are consolidation effort, wealth created or reserved and aggressive marketing efforts denoted as a vector with three elements [ *C*,*W*,*M*]. As an illustration of a duopoly system, we have two players (firms) represented as green (*g*) with strategy represented as [ *C*_*g*_,*W*_*g*_,*M*_*g*_] and yellow (*y*) with strategy represented as [ *C*_*y*_,*W*_*y*_,*M*_*y*_].
3. Determine the input and output variables of FISBD FIS: The inputs are market demand information (*D*) and production costs (*C*_*P*_) and the outputs are expected consolidation efforts (*E*_*c*_), expected wealth (*E*_*w*_) and expected aggressive marketing efforts (*E*_*m*_) where:
  *E*_*m*_ = 5 - (*E*_*w*_ + *Ec*) (Because the total (expected) resources of each player at any point is five).
4. Develop fuzzy sets, subsets and membership functions for all the input and output variables: This can be accomplished by soliciting knowledge from the experts or searching through literature data (Oderanti and De Wilde 2010). Sample membership functions for the output variable: *expected consolidation efforts* (*E*_*c*_) are as shown in Figure 3.

**Figure 3:**
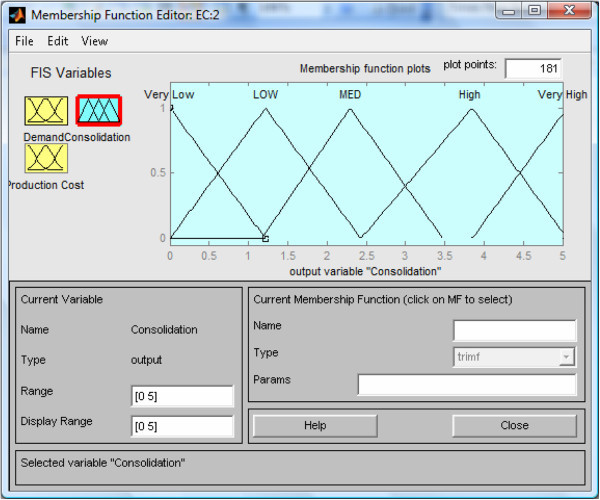
**FIS interface for the membership functions of the output variable** ***expected consolidation efforts*** **(** ***E***_***c***_ **) for the FISBD games.**
5. Formulate decision rules for the rule base: These also, ought to be solicited from experts (Oderanti and De Wilde 2010) and sampled rules are as shown in Figure 4.

**Figure 4:**
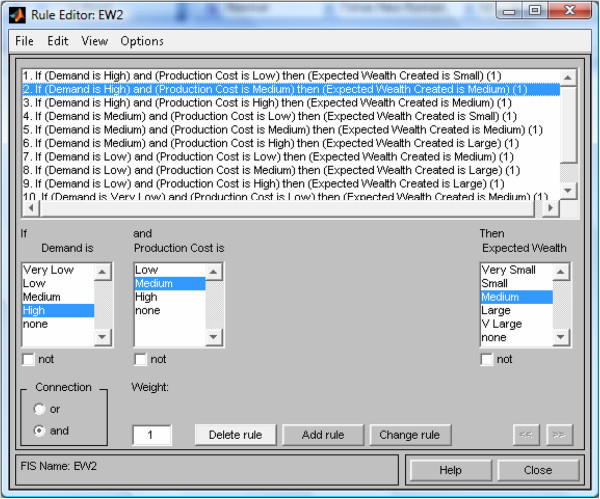
**Rule base with Matlab rule editor for** ***Expected Wealth*** **(** ***E***_***w***_ **) as output variable.**
6. Establish relationships between input values and their fuzzy sets and applying the decision rules. This could be accomplished through the use of fuzzy associative memory (FAM) tables (Oderanti and De Wilde 2010) and the fuzzy rule base can be coded into fuzzy inference system (FIS) using Matlab toolbox as shown in Figure 4 for the output variable *Expected Wealth* of the FISBD game.
7. Play the game: The procedure for playing the game is as follows: The game state is represented as vector [ *g*,*y*,*A*_*w*_,*r*]. *g* represents green player’s amount of resources, *y* represents yellow player’s amount of resources, *A*_*w*_ represents green’s accumulated wealth (profit) and *r* is the number of rounds the game is played. Green player strategy is denoted as [ *C*_*g*_,*W*_*g*_,*M*_*g*_] and yellow player strategy is denoted as [ *C*_*y*_,*W*_*y*_,*M*_*y*_] where: 1
  Because the total resources of each player at any point is five. As explained in Section "Players’ strategies", our choices of the number five in Equation 1 and for variable *r* are arbitrary. In a real system, any number that suitably represents the process can be chosen.
  General rules of the game are as follows:
   Initial stage of the game is [ 5,5,0,5] (i.e according to vector [ *g*,*y*,*A*_*w*_,*r*])
   At every state [ *g*,*y*,*A*_*w*_,*r*], green chooses his moves by allocating to his strategy [ *C*_*g*_,*W*_*g*_,*M*_*g*_] where *C*_*g*_ + *W*_*g*_ + *M*_*g*_ = *g* = 5 and yellow who is the fuzzy agent chooses his strategy [ *C*_*y*_,*W*_*y*_,*M*_*y*_] where *C*_*y*_ + *W*_*y*_ + *M*_*y*_ = *y* = 5.
   The game changes states as follows: 23456
    Where pence represents game payoff. Then, 7
    Because the total resources or expected resources of each player at any point is five. Now, 89
    Where *k* represents other costs which are taken to be zero to avoid needless complication (i.e *k* = 0). We define *E*_*w*_ (expected profit/Wealth)= *E*_*sp*_ - *C*_*P*_, where 10
    and *E*_*sp*_ represents the expected selling price of the product.
   The game ends when *r* = 0 and if *pence* is greater than zero (*pence* > 0), the green player wins, if less than zero (*pence* < 0), then the fuzzy agent player (yellow) wins else, the game is draw (i.e. if *pence* = 0).
   This 2-player game is a zero sum game and therefore, yellow loses whenever green wins and vice versa and since our aim is to develop an agent that would win as much as possible, maximize his payoff and minimize that of the opponents, Nash equilibrium (Abreu and Rubinstein 1988; Holt and Roth 2004) is not considered in this context.
8. Evaluate the fuzzy inference system (FIS): Using Matlab fuzzy toolbox, all the fuzzy inputs are passed into the Mamdani type FIS as shown in Figure 5.

**Figure 5:**
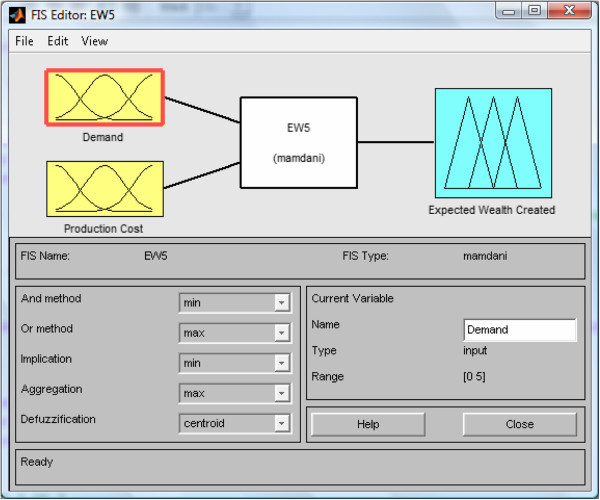
**Mamdani-type FIS interface for the FISBD games showing inputs** ***demand*** **(** ***D*** **) and** ***Production Cost*** **(** ***C***_***P***_ **) as well as expected wealth outputs (** ***E***_***w***_ **).**
9. Get the defuzzified output from the FIS: The crisp output for the FISBD is computed using centre of gravity method (COG) and sampled results are as shown in Figure 6 using rule view from Matlab FIS editor.

**Figure 6:**
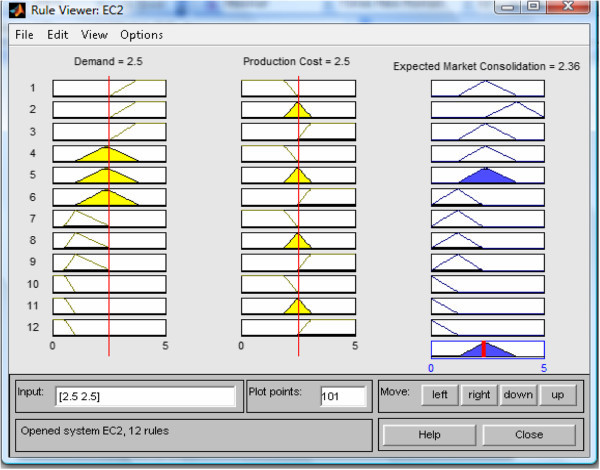
**Defuzzified (crisp) values for** ***expected market consolidation efforts*** ***Ec*** **at inputs** ***D*** **=** ***C***_***P***_ **= 2** ***.*** **5.**
10. Determine whether the conditions for the end of the game have been met: In this case study, the condition for the end of the game is when the number of rounds *r* reaches 1 counting down from 5 (i.e. when *r* = 1).
11. Training and performance evaluation: training and learning (Sudkamp and Hammell 1994) of the FISBD decision agent was accomplished through the optimization of the fuzzy logic parameters while using the game payoff as the basis for the performance measure after playing a series of the game as in (Braathen and Sendstad 2004; Oderanti and De Wilde 2010; Oderanti and Wilde 2011; Oderanti et al. 2012). This was achieved through the use of the *fminsearch* function in Matlab having considered other optimization algorithms such as gradient descent and genetic algorithm. *Fminsearch* uses the Nelder-Mead Simplex Search Method for finding the local minimum *x* of an unconstrained multivariable function *f*(*x*) using a derivative-free method and starting at an initial estimate.
  Consider two players G and Y playing the game, the expected outcome or payoff of a game can be denoted as *E*_*x*_(*G*,*Y*), using the notation of (Braathen and Sendstad 2004). As a training performance measure, the minimum expected payoff of an entire game taken over the class of all opponents *S* was used as in (Braathen and Sendstad 2004; Oderanti and Wilde 2011; Oderanti et al. 2012). If the fuzzy agent encounters the *strongest* opponent choice of strategy (say an opponent with strategy [0 0 5] as in iteration 12 in Table 1), the outcome of this play will result in a minimum payoff and the opponent may win the game. In (Braathen and Sendstad 2004; Oderanti and Wilde 2011; Oderanti et al. 2012), this very strict global performance measure was regarded as equity against globally optimizing opponent (*Geq*).
  11
  Another extreme opponent which may be regarded as weakest opponent will be that which reserves all his resources i.e. an opponent with strategy [0 5 0] (as in iteration 13 in Table 1) with respect to the strategic vector [ *C W M*], this results in FISBD fuzzy agent winning the game with highest payoff and we regard this as *equity against a locally optimizing opponent (Leq)*. 12
  These combined *global* and *local* performance measures are the basis for the rating of our FISBD decision agent.
  Meanwhile, a better optimization result may be achieved through *simulated annealing* (Kirkpatrick et al. 1983; Oderanti et al. 2012) but this is outside the focus of this research and may be considered as an avenue for further research.
  Furthermore, in this FISBD game, we do not employ a *maxmin* strategy but rather, we attempted to maximize the number of times that the fuzzy agent wins, and his payoff, while at the same time minimize those of the opponents.

**Table 1:** **Results of simulations of the untrained and trained agent in 2-player game**

Agent moves			Untrained		Control expt		Trained	
S/N	Green	Yellow	Winner	Payoff	Winner	Payoff	Winner	Payoff
1	3, 1, 1	2, 0, 3	Yellow	-289.3	Yellow	-192.9	Yellow	-305.0
2	0, 5, 0	1, 4, 0	Yellow	-99.8	Yellow	-66.6	Yellow	-142.2
3	0, 5, 0	0, 1, 4	Yellow	-704.8	Yellow	-469.9	Yellow	-747.2
4	4, 0, 1	4, 0, 1	Green	40.8	Green	61.3	Yellow	-8.2
5	1, 0, 4	2, 0, 3	Green	351.6	Green	527.5	Green	302.2
6	3, 1, 1	4, 0, 1	Yellow	-16.1	Yellow	-10.7	Yellow	-65.2
7	3, 0, 2	2, 1, 2	Green	136.8	Green	205.2	Green	94.9
8	3, 1, 1	3, 1, 1	Green	14.8	Green	22.34	Yellow	-34.2
9	1, 1, 3	1, 0, 4	Yellow	-22.0	Yellow	-14.7	Yellow	-63.9
10	2, 1, 2	1, 1, 3	Yellow	-52.7	Yellow	-35.2	Yellow	-94.8
11	3, 0, 2	2, 0, 3	Yellow	-26.7	Yellow	-17.9	Yellow	-68.8
12	0, 0, 5 (*Geq*)	0, 5, 0 (*Leq*)	Green	1054.5	Green	1581.8	Green	1012.0
13	0, 5, 0 (*Leq*)	0, 0, 5 (*Geq*)	Yellow	-863.8	Yellow	-575.9	Yellow	-906.2

From the table, the first column shows the serial numbers of the iterations, the second column contains player *green’s* strategies while the third column contains that of *yellow*. For example, in the fifth iteration, *green’s* strategy shows [1, 0, 4] this indicates how resources are allocated to strategy [*C,W,M*]: *C* = 1, *W* = 0 and *M* = 4. The forth column gives the winners for the untrained simulations while the fifth column gives the payoffs of those simulations. Column six and seven show the winners for the *control experiment*. The control experiments show the results where both players did not use fuzzy inference systems in playing the games. Column eight and nine show the winners for the *trained* simulations. These results show that the fuzzy player (**Yellow**) was able to win more than the competitor (**Green**) because he made use of the fuzzy inference system in making his business decisions. Also, it can be observed that the trained agent is able to perform better after training. The minus sign on yellow payoffs merely shows zero-sum. The strongest opponents (*Geq*) and weakest opponents (*Leq*) are shown in iterations 12 and 13 respectively.

**Figure 7:**
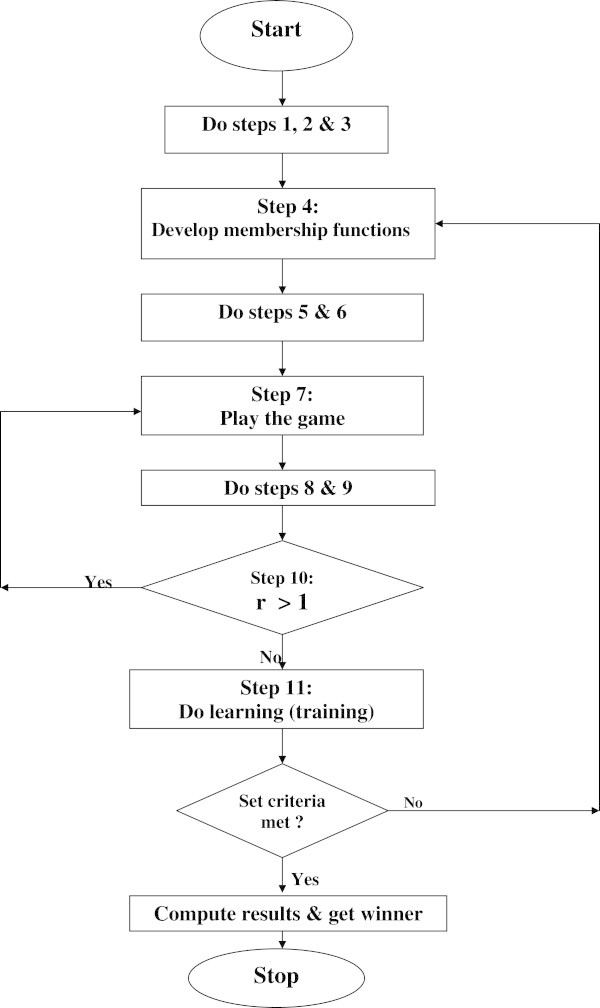
**Chart showing the two loops of the wage game.** The first loop stops when *r* = 1 (this means the fifth round of the game) and the second loop represents learning of the fuzzy player and it stops when the set performance criteria have been met as explained in step 11.

All these steps that are necessary for playing this game are as summarized in the flowchart shown in Figure 7.

## Procedures for *n*-player game

In this section, we examined *n*-player games that represent perfect market competitions with many players. Our fuzzy player is still represented as *yellow*, the *n*-th player, who faces *n* - 1 opponent players (competitors). The *n*-player games also follow the procedural steps of 2-player FISBD general illustrations in Section "Fuzzy inference system for business decisions (FISBD)" with exceptions to steps 2 and 7 which are modified as follows:

-  Step (2) Determining the strategy: as an example of a perfect market competition, we have *n* players. For *j* = 1 to *n* - 1, the opponents *P*(*j*) strategies are denoted as [ *C*(*j*),*W*(*j*),*M*(*j*)] and the fuzzy agent (yellow) strategy as [ *C*(*n*),*W*(*n*),*M*(*n*)].
-  Step (7) Play the game: procedures for playing the game are as follows: The game state is represented as vector *S* = [ *P*_1_,*P*_2_,⋯,*P*_*n*-1_,*P*_*n*_,*A*_*w*_,*r*]. Where *P*_1_ to *P*_*n*-1_ represent opponent players’ (competitors) amount of resources, *P*_*n*_ represents fuzzy agent player (yellow) amount of resources, *A*_*w*_ represents opponents’ accumulated wealth (profit) and *r* is the number of rounds the game is played. Both the competitors and fuzzy player strategy are as stated in step 2 above. 13
  As explained in Section "Players’ strategies", our choices of the number five in Equation 13 and for variable *r* are arbitrary. In a real system, any number that suitably represents the process can be chosen.
  General rules of the game are as follows:
  -  Initial state of the game is [ 5,5,⋯,5,5,0,5] according to the vector [ *P*_1_,*P*_2_,···,*P*_*n*-1_,*P*_*n*_,*A*_*w*_,*r*].
  -  At every state [ *P*_1_,*P*_2_,⋯,*P*_*n*-1_,*P*_*n*_,*A*_*w*_,*r*], for *j* = 1 to *n* - 1, the opponents *P*(*j*) choose their moves (strategies) [ *C*(*j*),*W*(*j*),*M*(*j*)] where: *C*(*j*) + *W*(*j*) + *M*(*j*) = *P*(*j*) = 5 and yellow who is the fuzzy player chooses his strategy [ *C*(*n*),*W*(*n*),*M*(*n*)].
  -  The game changes states as follows: 14
    Where W(n) is the fuzzy agent’s wealth 15161718
  -  The game ends when *r* = 0 and if *pence* is greater than zero, (*pence* > 0), then one of the opponent players wins, if less than zero (*pence* < 0), then the fuzzy agent player (yellow) wins else, the game is a draw (i.e. if *pence* = 0).
  -  The rest of the procedures follow those steps highlighted in Section "Fuzzy inference system for business decisions (FISBD)" above for the 2-player FISBD game.

## Results discussion for 2-player and n-Player Games

**Figure 8:**
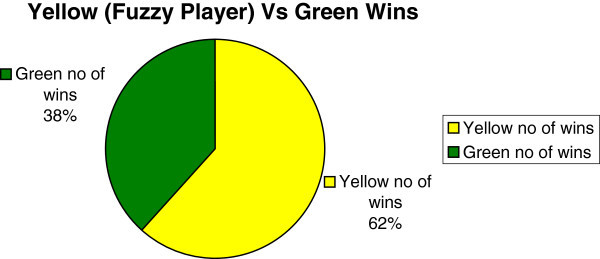
**Results show that the fuzzy player (yellow) wins more often than the competitor (Green) because he made use of the fuzzy inference system (FIS) in making his business decisions from the results in Table** 1 **.**

Sampled results of a typical 2-player FISBD experiment in accordance with the procedure highlighted above are as shown in Table 1. The pie chart in Figure 8 and data on Table 1 show that the fuzzy player (**Yellow**) was able to win more than the competitor (**Green**) because he made use of the fuzzy inference system in making his business decisions.

From equations 4 and 5 and from the results in Table 1, it will be seen that for any of the players to win the game, he must allocate a substantial part of his resources to aggressive marketing and this allocation must outweigh that of the opponent’s allocation.

According to this model and with respect to the two equations, since the number of rounds *r* decreases as the game is played, this reduces the strength of marketing aggressiveness. An entrepreneur who is a new entrant into an industry, is best advised to try as much as possible to devote much of his resources on aggressive marketing campaigns (*M*) than other strategies (i.e. efforts on consolidation (*C*) and reserved wealth (*W*)). This will enable him to have a strong footing in the industry and to be able to have a large market share as early as possible as the game is played and thus, will result in winning the game.

However, because the fuzzy player is able to capture the uncertainty in the business environment more effectively and efficiently as a result of the fuzzy rules in the fuzzy inference system, he is able to override the system and wins more often than the opponent. From the results in column four and five of Table 1, out of thirteen iterations shown on the table, the fuzzy player (yellow) wins in eight iterations (iterations 1, 2, 3, 6, 9, 10, 11 and 13) while the opponent (green) wins in only five iterations which are iterations 4, 5, 7, 8 and 12.

As shown in columns six and seven of Table 1, we verified these results by designing control experiments (simulations) in which the fuzzy player does not change his moves in accordance with the fuzzy rule base. The results obtained from the control experiments show that the game follows conventional trends, that is, the fuzzy player wins only where he allocates more units of resources to his marketing strategy at the start of the game than those of his competitors and his payoff also depends on this. The payoff of the fuzzy player in the control experiments (where he did not use fuzzy rule base) are far less than what he got when he used fuzzy rule base to make his business decisions.

**Figure 9:**
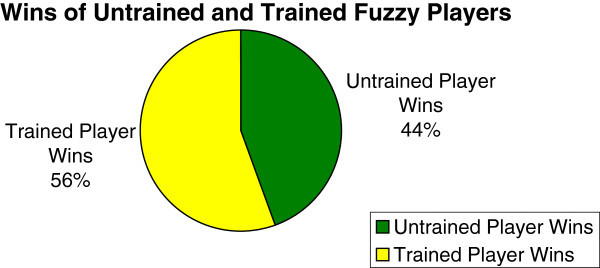
**This chart shows how the performance of the fuzzy player increased after training as it won more often than it won before training from the results in Table** 1 **.**

Moreover, after learning, as stated in Section "Fuzzy inference system for business decisions (FISBD)" Step 11 and as shown in Table 1, the fuzzy player performs much better as the agent was able to win more than he won before training. Figure 9 compares the number of fuzzy wins before and after training.

Results in columns eight and nine show the the performance of the players after learning (training). The columns show that after learning, out of the same thirteen iterations that were used before learning, the fuzzy player wins a total of ten iterations (additional wins of two iterations and these therefore means losses to the opponent) while the opponent wins only three iterations. After learning, the two additional iterations won by the fuzzy player, as shown in the table, are iterations 4 and 8. Therefore, these means that the opponent has lost two additional iterations as a result of zero sum concept.

A typical example is when the two players chose [ 4,0,1] and when they both chose [ 3,1,1]. In both cases and some other cases, before training, it was green that won the game while after training, it was the fuzzy agent (yellow) that won. Moreover, in all cases, even when green wins, his payoff (*pence*) is always smaller (minimized) after learning of the fuzzy agent than what it was before learning.

For examples, in iterations 5, 7 and 12 (the only three iterations where green player wins after learning), before leaning of the fuzzy player, green’s payoffs were 351.6, 136.8 and 1054.5 for those three iterations respectively. However, after learning, these were reduced (minimized) by the **learned fuzzy player** and therefore, green’s payoffs for those iteration become 302.2, 94.9, and 1012.0 respectively.

From the results explained above, it can be observed that training (learning) of the fuzzy agent was really important and the training algorithm was very effective because it enables the agent to learn and reach the performance criteria.

At the end of the game, the estimated price for the commodity can be forecast with Equation 10: (*E*_*sp*_ = *E*_*w*_ + *C*_*P*_).

From the *n*-player simulations shown in Table 2, it was observed that because of the ability of the fuzzy player to grasp effectively the uncertainty in the business environment by changing his strategy based on the information provided by the fuzzy rule base, the fuzzy player wins more often as the number of competitors (opponent players) increases.

**Table 2:** **Results of simulations of n-player game when** ***n*** **=3**

Agent moves				Untrained player		Control expt		Trained agent	
S/N	Green	Brown	Yellow	Winner	Payoff	Winner	Payoff	Winner	Payoff
1	3, 1, 1	0, 1, 4	2, 0, 3	Yellow	-26.5	Yellow	-17.7	Yellow	-95.8
2	0, 5, 0	0, 5, 0	1, 4, 0	Yellow	-117.1	Yellow	-78.1	Yellow	-182.6
3	0, 5, 0	0, 0, 5	0, 1, 4	Yellow	-243.6	Yellow	-162.4	Yellow	-309.9
4	4, 0, 1	4, 0, 1	4, 0, 1	Yellow	-4.5	Yellow	-3.0	Yellow	-72.7
5	1, 0, 4	3, 2, 0	2, 0, 3	Yellow	-138.5	Yellow	-92.3	Yellow	-205.4
6	3, 1, 1	3, 0, 2	4, 0, 1	Green	82.9	Green	124.4	Green	22.3
7	3, 0, 2	2, 0, 3	2, 1, 2	Green	235.4	Green	353.1	Green	170.5
8	3, 1, 1	3, 1, 1	3, 1, 1	Yellow	-34.5	Yellow	-23.0	Yellow	-102.7
9	1, 1, 3	1, 1, 3	1, 0, 4	Yellow	-59.1	Yellow	-39.4	Yellow	-128.2
10	2, 1, 2	2, 1, 2	1, 1, 3	Yellow	-96.4	Yellow	-64.3	Yellow	-163.4
11	3, 0, 2	0, 4, 1	2, 0, 3	Yellow	-328.1	Yellow	-218.8	Yellow	-385.8
12	0,0,5	0, 0, 5	0, 5, 0	Green	1397.4	Green	2096.1	Green	1330.7
13	0,5,0	0, 5, 0	0, 0, 5	Yellow	-1145.1	Yellow	-763.4	Yellow	-1210.6

From the table, the first column shows the serial numbers of the iterations, the second column contains player *green’s* strategies, third column contains those of player *brown*, while the forth column contains that of *yellow*. For example, in the fifth iteration, *green’s* strategy shows [1,0,4], this indicates how resources are allocated to strategy [*C,W,M*]: *C* = 1, *W* = 0 and *M* = 4. The fifth and sixth column gives the winners and payoffs for the untrained simulations. It can be observed that the fuzzy player performs better than it does in 2-player game results shown on Table 1. For example, in iterations 1, 3 and 5 where one of the opponents allocated higher strategy to marketing which is the strongest strategy, one expects the fuzzy player to lose but it won. It also happened in many other iterations which are not shown here for lack of enough space. Also, the fuzzy player has higher payoffs than in 2-player game.

**Figure 10:**
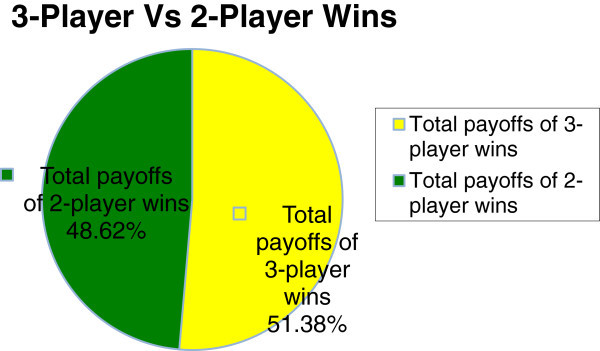
**This chart compares the total payoffs of the fuzzy (yellow) player in both 2-player (column 5 of Table** 1 **) and 3-player (column 6 of Table** 2 **) games.** These trends continue for (*n* = 100) players and more.

As shown in Table 2 and Figure 10 with three players (*n* = 3), very interesting cases are seen in those iterations where one expected the fuzzy player to lose because he started the game with weaker strategies than those of his competitors (as it happens in 2-player games), but because the player reasons in accordance with the fuzzy engine (rule base) and changes his strategies accordingly, the fuzzy player wins in those cases, and better than he wins in the 2-player game results shown on Table 1.

**Figure 11:**
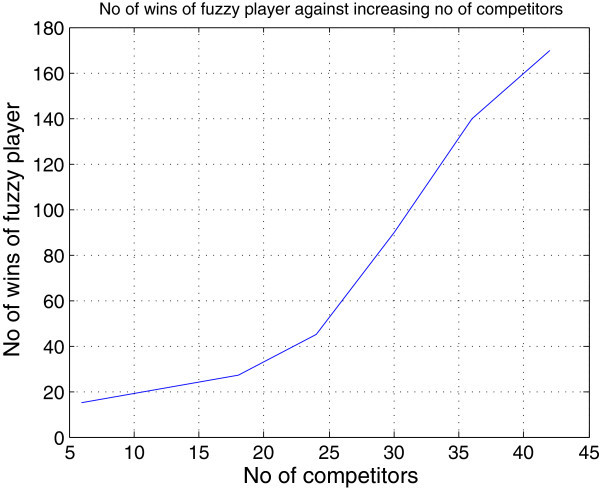
**A graph showing the strength of the fuzzy player with respect to increasing number of competitors: It can be observed that the fuzzy player performance in the games improve as the number of competitors (opponent players) increases.**

This shows that the fuzzy player performs better in the 3-player game than in the 2-player games where he won only eight iterations out of thirteen iterations as shown in Table 1. This shows that because of the fuzzy inference reasoning being used by the fuzzy player, the more the number of competitors, the better the payoffs of the fuzzy player. These trends continue with large number of competitors and after running several simulations with the number of players *n* ranging from 1 to 100, the results of the *n*-player FISBD game show that the larger the number of opponent players (competitors), the better the fuzzy player performs, as illustrated in graphs of Figure 11 and Figure 10 (with up to fifty competitors), due to the fact that he is able to adequately capture the uncertain information at his disposal which was modelled using the concepts of fuzzy reasoning.

## Conclusion

We have modelled decision making processes under uncertainty in business games, using fuzzy logic concepts and game theory. Our model was termed fuzzy inference system for business decisions (FISBD). We illustrated this for 2-player games that represent duopoly market structure and *n*-player games that represent perfect market structure. A fuzzy inference system for business decisions was designed and implemented using Matlab software. Fuzzy rules were constructed in developing the FISBD model using the Matlab toolbox and the implementation of this model heavily depends on expert knowledge and experience to facilitate the development of a reasonable fuzzy rule base for the determination of the if-then rules that denote the relationship between inputs and the output variables.

Furthermore, we have applied a learning algorithm to the decision processes which enables the decision agent to optimize his performance in the decision processes as the games were played so as to meet the set criteria. To do the learning, the Nelder-Mead simplex method for finding the minimum of an unconstrained multivariable function was used.

Results of the learning showed that the learning algorithm works very effectively and efficiently as the fuzzy player (yellow) was able to perform much better after learning with higher payoffs and this enables him to reach the set criteria

We verified these results by designing a control experiment (simulation) in which the fuzzy player does not change his moves in accordance with the fuzzy rule base. The payoff of the fuzzy player in the control experiment (where he did not use fuzzy rule base) are far less than what he got when he used fuzzy rule base to make his business decisions.

Our FISBD procedure has practical uses in business contexts as it can serve as very useful tools in the hands of an entrepreneur to:

-  Advise him on certain marketing strategic decision policies that can keep his business in strategic advantage over his competitors in the market.
-  Give him insight on how his firm can successfully compete with its peers in the market by determining how much of its available resources or efforts could be dissipated on our three adopted strategies of marketing in such a way that his profit (accumulated wealth) will be maximized.
-  Effectively utilize the uncertain (fuzzy) and prevailing or anticipated market demand (*D*) information, cost of producing a commodity (*C*_*P*_) and other fuzzy information at his disposal to achieve the set goal of his business.

Also, we have been able to supplement the laws of demand and supply with a more practical approach which takes into consideration the uncertain (fuzzy) nature of most information available to business decision makers. While the traditional laws of demand and supply address the nature of decision processes by consumers and suppliers respectively, our own approach extends them further. This is to address the nature of decision processes by an intending entrepreneur or manufacturer to forecast the prospect of the proposed business through profit prediction from estimated selling price given the fuzzy market or industry information available to him. This allows him to determine price and marketing strategies in function of a very low, medium, high, very high, etc. demand.

We have demonstrated that our model works well with large number of players. We illustrated this by using *n*-player games that represent perfect market structure. The results of our *n*-player simulations showed that an entrepreneur needs not to worry about the proliferation of competitors in the industry because by adopting this FISBD model, the results of the *n*-player games show that the larger the number of opponent players (competitors), the better the fuzzy player performs in the games as shown in Table 2 and Figure 11.

In arriving at our results, the simulations are based on assumptions and conditions that the players involved in the decision processes are rational players (Section "Introduction") and that only the fuzzy player (yellow), at the moment, uses fuzzy moves (Zhang and Kandel 1997). This is in accordance with our overall aim of designing models that illustrate how an entrepreneur could make effective and efficient business decisions by using fuzzy inference systems (FIS) in capturing uncertainties that may surround his business environments. This will therefore help the entrepreneur to have competitive advantages over his competitors who are unaware of the usefulness of these tools and therefore are not making use of the fuzzy inference models in their decision making processes.

These models can be used as effective and efficient decision tools by business organisations that are operating in different scenarios similar to those we have described in this paper. However, in using the models as decision tools, the entrepreneur will need to adapt, adjust and modify the variables and the decision rules to suit the situations in question as well as his business environments.

For example, rather than competing with capital resources (say £5M), the organisation’s competing resources may be in terms of roles assigned to personnels in the organisation. For instance, due to persistent reduction in sales over the last few weeks, an organisation may decide to assign more personnels to the marketing department (*M*) and less to the operation department (*C*) of the organisation. The organisation will then change these roles until desirable results are attained in the business.

We have used general methodology and illustration to describe the model in this paper and we have verified the validity of our results and methodology with more case studies and real data in our other papers in (Oderanti and De Wilde 2010; Oderanti and Wilde 2011; Oderanti et al. 2012). The papers contain different business scenarios that entrepreneurs encounter in day to day business operations.

## Future research

We will apply this model in a wider range of micro and macroeconomic models that are targeted to specific industries and international trades among countries.

Experiments may be carried out to determine the actual duration and number of steps in the business games. In our model, we arbitrarily chose the steps based on expert advice and from the game experiments in (Braathen and Sendstad 2004). However, further work may be carried out to determine the actual duration for the business games.

To replace the adaptation of the membership functions by operations on type-2 fuzzy sets (Méndez and Hernandez 2008). Type-2 fuzzy sets address the issues concerning uncertainty about the value of the membership functions and it allows incorporating uncertainty about the membership function into fuzzy set theory (Mendel 2007).

Also, the model can be applied for optimizing bidding in auctions and other areas of economics such as trading.

This automatic decision system can also be extended to capture human activities where available data are mostly uncertain or fuzzy such as in meteorology or weather forecasting and in designing embedded systems (Gajski and Vahid 1995) for business enterprises.

Future work of this nature can also be channelled toward applications in robotics which is an area in artificial intelligence that is concerned with the practical uses of robots. A robot is a machine that is guided automatically and that is capable of doing tasks on its own. One of the other major characteristics of a robot is that by its movements or appearance, it often conveys a sense that it has intent or agency of its own. Therefore, fuzzy logic concepts and game theory may be introduced to integrate further intelligence into robots to enable them capture and grasp various uncertain events in their movements.

Learning (training) of the fuzzy system will also help robot to learn in making better decisions using fuzzy inference systems and also to deal with systems requiring advanced decision making in unpredictable environments (Saridis 1983).

Also, future work may be carried out to test the system behaviour toward other fuzzy inference techniques. In our model, we have used Mamdani-type fuzzy inference system. However, there are other inference techniques that can be tested on the system and evaluate its performance. other popular common methods of deductive inference for fuzzy systems (Ross 2005) that can be tested on this model are:

-  Sugeno systems
-  Tsukamoto models

Other areas of future work may also be channelled toward trying other optimization algorithms on the system and evaluate the performance of the models. Other optimization techniques that may also be tried on the models.
